# Interactions between rainfall, deforestation and fires during recent years in the Brazilian Amazonia

**DOI:** 10.1098/rstb.2007.0026

**Published:** 2008-02-11

**Authors:** Luiz Eduardo O.C Aragão, Yadvinder Malhi, Nicolas Barbier, Andre Lima, Yosio Shimabukuro, Liana Anderson, Sassan Saatchi

**Affiliations:** 1 Environmental Change Institute, Oxford University Centre for the Environment, University of OxfordOxford OX1 3QY, UK; 2 Université Libre de Bruxelles, Service de Botanique Systématique et PhytosociologieCP 169, 1050 Bruxelles, Belgium; 3 Brazilian Institute for Space Research (INPE), São José dos CamposSão Paulo 12227-010, Brazil; 4 Jet Propulsion Laboratory, California Institute of TechnologyPasadena, CA 91109, USA

**Keywords:** Amazonia, fire, deforestation, drought, land use, climate change

## Abstract

Understanding the interplay between climate and land-use dynamics is a fundamental concern for assessing the vulnerability of Amazonia to climate change. In this study, we analyse satellite-derived monthly and annual time series of rainfall, fires and deforestation to explicitly quantify the seasonal patterns and relationships between these three variables, with a particular focus on the Amazonian drought of 2005. Our results demonstrate a marked seasonality with one peak per year for all variables analysed, except deforestation. For the annual cycle, we found correlations above 90% with a time lag between variables. Deforestation and fires reach the highest values three and six months, respectively, after the peak of the rainy season. The cumulative number of hot pixels was linearly related to the size of the area deforested annually from 1998 to 2004 (*r*^2^=0.84, *p*=0.004). During the 2005 drought, the number of hot pixels increased 43% in relation to the expected value for a similar deforested area (approx. 19 000 km^2^). We demonstrated that anthropogenic forcing, such as land-use change, is decisive in determining the seasonality and annual patterns of fire occurrence. Moreover, droughts can significantly increase the number of fires in the region even with decreased deforestation rates. We may expect that the ongoing deforestation, currently based on slash and burn procedures, and the use of fires for land management in Amazonia will intensify the impact of droughts associated with natural climate variability or human-induced climate change and, therefore, a large area of forest edge will be under increased risk of fires.

## 1. Introduction

There is a growing concern about the impacts of climate change on the stability of ecological processes in Amazonia, the resulting feedbacks from the local to the global circulation system and the ensuing consequences on plant, animal and human populations. Some global circulation models suggest that Amazonia may be vulnerable to extreme drying in response to circulation shifts induced by global warming (Li *et al.* 2006), possibly leading to a dieback of tropical rainforest with potential acceleration of global warming (Cox *et al.* 2004).

Amazonian droughts have been strongly related to El Niño events, such as in 1982/1983, 1986/1987 and 1997/1998 (Marengo 1992; Uvo *et al.* 1998; Ronchail *et al.* 2002; Marengo 2004) and more recently to the tropical Atlantic sea surface temperature (SST) anomalies associated with the Atlantic Multidecadal Oscillation (Li *et al.* 2006; Good *et al.* 2008; Marengo *et al.* in press). The increase of the tropical Atlantic SST alone has been implicated as a causal factor of the severe drought that affected Amazonia in 2005 (Marengo *et al.* in press).

The impacts of reducing rainfall over Amazonia are likely to be exacerbated by the synergic interactions among other anthropogenic forcing factors such as deforestation and fires (Cochrane & Laurance 2002; Hutyra *et al.* 2005). Positive feedbacks among deforestation, fires and drought have been previously reported (e.g. Cochrane *et al.* 1999; Laurance & Williamson 2001). Drought alone is reported to reduce tree growth, increase tree mortality (particularly in forest edges) and increase leaf shedding. This process leads to the increase of canopy openness and understorey insolation with consequent drying of the accumulated litter. When these conditions are combined with intense forest degradation through edge effects and logging, the risk of forest fires can increase dramatically in Amazonia (Uhl & Kauffman 1990; Cochrane & Schulze 1999; Cochrane *et al.* 1999; Laurance & Williamson 2001; Barlow & Peres 2004; Nepstad *et al.* 2004). On the other hand, large-scale forest conversion (Nobre *et al.* 1991; Laurance & Williamson 2001; Laurance *et al.* 2002; Silva Dias *et al.* 2005; Costa *et al.* 2007) and the smoke from fires (Rosenfeld 1999; Ackerman *et al.* 2000; Artaxo *et al.* 2005) may promote a reduction in rainfall over these areas. This chain of events generates a positive feedback loop that increases the vulnerability of Amazonia to climate change.

In this study, we focus on Brazilian Amazonia where data are readily available. Here we used satellite-derived time series of rainfall, fires and deforestation to explicitly quantify the seasonal patterns of these three variables and their relationships, with a particular focus on the 2005 Amazonian drought. In addition, we investigate how rainfall and deforestation influence fire dynamics at the monthly and annual time scales. Finally, we discuss how climate variability and the occurrence of droughts, deforestation and fires can potentially increase the vulnerability of Amazonia to climate change.

## 2. Material and methods

### (a) Rainfall, fire and deforestation datasets

We used a time series (January 1998–December 2006) of cumulative monthly precipitation (mm per month) derived from the tropical rainfall measuring mission data (TRMM 3B43-v6) at 0.25° spatial resolution (NASA 2006). The validation of this dataset showed that TRMM product captures the rainfall patterns of the Amazonian region accurately (Aragão *et al.* 2007).

The INPE-DETER (Detection of Deforested Areas in Real Time) dataset (INPE 2006*a*) was used to quantify the cumulative monthly area (km^2^) of deforested polygons (April 2004–October 2005 and March 2006–September 2006). Deforestation values for four missing months (November 2005–February 2006) were estimated using proportional values between the subsequent months in the previous year. In addition, the time series (1998–2005) of annual cumulative deforested area was obtained from the INPE-PRODES (Assessment of Deforestation in Brazilian Amazonia) dataset (INPE 2005).

Hot pixel counts were derived from daily, 1 km spatial resolution, NOAA-12 (National Oceanic and Atmospheric Administration) database from the Brazilian Institute for Space Research (INPE) *Queimadas* project (mid-1998–2005; INPE 2006*b*). Hot pixels are indicators of fires and may well underestimate their occurrence owing to clouds and forest canopy cover, but hot pixel counts do allow the evaluation of patterns over time.

### (b) Data analysis

We extracted from the remote-sensing surfaces the monthly cumulative values of the area of deforested polygons and the number of hot pixels as well as the average rainfall within the limits of the Brazilian Legal Amazonia (approx. 4 000 000 km^2^). This region includes the states of Amazonas, Acre, Rondônia, Roraima, Mato Grosso, Pará, Amapá, Maranhão and Tocantins.

We analysed the behaviour of the monthly rainfall, deforested area and hot pixels through time to identify possible seasonality in the data. As an additional support for the interpretation of these data, we generated four maps showing first the total cumulative deforestation in Amazonia, based on INPE-DETER data, and subsequently the hot pixels and counts in 2005 for each one of the three land cover classes defined in the deforestation map. Time series were analysed and compared using (cross)-spectral analysis (Priestley 1981; Diggle 1989). This well-established approach extends the power spectra methodology to the comparison of pairs of time series. The values in a power spectrum, computed as the squared amplitude of the Fourier transform of the signal, correspond to the breaking down of the signal's variance into frequency bins. In other words, the relative strength of a periodic component of a given frequency in the signal is given by the power spectrum value at that frequency. For a pair of signals, one can equivalently compute a combined power spectrum (or cross-spectrum), which allows exploring shared periodicities (cycles yr^−1^) between the two signals. In this case, it is the covariance between signals, which can be investigated at specific temporal scales (frequencies). The resulting cross-spectrum can be analysed in terms of amplitude and phase. The coherence spectrum, which is the amplitude normalized between 0 and 1, can be interpreted as a Pearson product–moment correlation coefficient between series, computed for each frequency. The phase spectrum indicates the phase lag between signals. A strong coherence for a specific temporal frequency, combined with a null phase shift, indicates a positive correlation while a phase shift of *π* corresponds to a negative correlation. Variance estimates can be computed for both coherence and phase spectra (Diggle 1989), to allow building pointwise CIs for these estimates.

After identifying the connections among the variables, we conducted a regression analysis using the monthly and annual data to explore the shape of the relationship between the variables.

## 3. Results and discussion

### (a) Seasonality of rainfall, fire and deforestation and their relationships

Results presented in figure 1 show that rainfall, deforestation and hot pixels have a marked annual periodicity. Our analysis indicates that, on average, the dry season (rainfall below 100 mm per month, based on Aragão *et al.* 2007) persists from July to September for most of the years analysed, excepting 2005, when the dry season started in June (figure 1*a*), in association with the drought that struck the basin in this year. Both deforestation and hot pixels peaked during the dry season in Amazonia. The major peak of deforestation is observed in May (figure 1*b*) while the peak of hot pixels coincides with the months of lowest rainfall, August and September (figure 1*c*).

The seasonal signal of fires observed here can be decomposed into three distinct types: (i) areas that have been deforested and then burnt in the same year, (ii) areas that have been deforested in previous years and then burnt later, and (iii) fires in natural vegetation and other non-forest areas that are not included in the INPE dataset. To investigate the relative contribution of each of these categories to the total number of hot pixels observed, the 2005 map of hot pixels was overlaid on the deforestation map derived from INPE-DETER data and subdivided into land cover classes (figure 2).

Our results demonstrate that fires in deforested areas contributed to 60% of the total number of hot pixel detections in 2005. Of the remaining 40% of detections, 28% occurred in forests and 12% in areas considered as non-forest in the INPE land cover classification. The hot pixels in areas deforested during 2005 and until 2004 contributed to 8 and 92%, respectively, of the total number of detections in deforested areas. On the other hand, the large percentage of hot pixels detected in forests during 2005 was associated with the leakage of fires from the deforested areas to surrounding drought-stressed forest edges (Aragão *et al.* 2007).

The spectral analysis of the monthly data stressed a clear seasonal variation in all time series analysed (figure 3*a*–*c*), as all power spectra showed a peak for a frequency of one cycle yr^−1^. Deforestation, however, is dominated by another peak at two cycles yr^−1^.

The coherence spectra (figure 3*d*–*f*) showed that the correlation among all time series was higher than 90% for an annual periodicity (i.e. one cycle yr^−1^). Focusing on the coherent annual frequency (highlighted by an arrow in figure 3*g*–*i*), the phase spectrum showed a phase shift of approximately *π*/2 for the relation between hot pixels and deforestation, meaning that deforestation led the presence of hot pixels by approximately three months (note that at one cycle yr^−1^, 2*π*, *π* and *π*/2 are equivalent to 12, 6 and 3 months time lag, respectively). Similarly, the relationship between rainfall and deforestation had a phase shift of approximately *π*/2, which indicates that the peak of rainfall precedes the deforestation peak by three months; however, the second deforestation peak highlighted in the power spectra coincides with the peak of the dry season. Finally, the comparison between rainfall and hot pixels revealed, as expected, that rainfall was negatively correlated (phase shift of *π*) with the number of hot pixels. Therefore, the peak of hot pixel detections matches the peak of the dry season in Amazonia without time lag.

These results elucidated the interaction between climate and land-use practices, describing the timing of slash and burn activities in the Brazilian Amazonia. In summary, approximately three months after the peak of the rainy season, deforestation reaches its highest annual values. In this case, there is a prognostic action in relation to the peak of the dry season, giving time for the fallen wood to dry until the driest month. Afterwards, during the peak of dry season (minimum rainfall values), farmers set fire to the dry material on the ground and hot pixel values reach their maximum.

### (b) Influence of monthly and annual rainfall and deforestation on fire dynamics

At the monthly time scale, deforested area increases exponentially with the decrease of rainfall assuming the three-month lag defined in the spectral analysis (*r*^2^=0.74, equation (3.1); figure S1 in the electronic supplementary material).

(3.1) $$\text{deforestation}=4116.55\;{\text{exp}}^{-0.01\times \text{rain}}.$$

This means that the higher rate of deforestation in April/May is strongly related to the rainfall in June/July, which is the beginning of the dry season in most of the Brazilian Amazonia. Besides this, hot pixel detections tended to increase exponentially with the decrease of rainfall (*r*^2^=0.60, equation (3.2); figure S1 in the electronic supplementary material)

(3.2) $$\text{hot}\;\text{pixels}=63588.\text{21}\;{\text{exp}}^{-0.02\times \text{rain}}.$$

Conversely, we did not find a strong relationship between hot pixels and deforested area at the monthly time scale.

Despite the fact that deforestation may not be a major predictor of hot pixel counts, either spatially (Cardoso *et al.* 2003) or temporally at a monthly scale, we found a strong linear relationship (*r*^2^=0.84, *p*=0.004, equation (3.3)) between the annual cumulative number of hot pixels and the size of the area deforested annually from 1998 to 2004

(3.3) hotpixels=8.50×deforestation−69570.59.

We attributed the linear trend observed between hot pixels and deforestation from 1998 to 2004 to the expansion of pastures for cattle ranching and large areas of mechanized agriculture (Morton *et al.* 2006) in the southern part of Brazilian Amazonia. The expansion of mechanized agriculture was mainly driven by the area planted by soya bean crops in Amazonia that increased from 1×10^6^ ha in 1990 to 7×10^6^ ha in 2005, with an expansion rate of 17% yr^−1^ from 2000 to 2005 (Costa *et al.* 2007). However, during the 2005 drought, the effect of rainfall deficit overtook the influence of land-use change on hot pixel dynamics (figure 4).

### (c) Interactions between land-use and climate change and the vulnerability of Amazonia

In the last decade Amazonia experienced two droughts, in 1997/1998 and 2005. Both droughts caused significant rainfall anomalies and hydrological stress, significantly increasing the number of fires detected over this region (Aragão *et al.* 2007). The areas affected by fires are expected to become more vulnerable to recurrent fires (Uhl & Kauffman 1990; Cochrane & Schulze 1999; Nepstad *et al.* 1999).

The interaction between land-use and climate change is likely to generate a positive feedback (e.g. Cochrane *et al.* 1999), increasing the vulnerability of Amazonia to climate change, and have significant effects on the global carbon cycle. For example, the estimated global flux of CO_2_ to the atmosphere from land-use change was 1.6 (0.5–2.7) Pg C yr^−1^ for the 1990s, 22% of total anthropogenic emissions (Denman *et al.* 2007). The Brazilian Amazon alone might yield a net flux of carbon from the biosphere to the atmosphere of 0.1–0.4 Pg C yr^−1^, due to land-use change (Houghton *et al.* 2000). This is equivalent to 6–25% of the total carbon emissions from land-use changes. These emissions can overtake the sink of carbon calculated for the undisturbed ecosystems in this region (Nepstad *et al.* 1999; Barlow & Peres 2004; Malhi & Phillips 2004; Phillips *et al.* 2008).

The effect of deforestation on fire impacts is likely to be exacerbated by drought events, which may become more frequent under some climate change scenarios (Timmermann 1999; Cox *et al.* 2004; Li *et al.* 2008). Based on the relationship found between deforested area and hot pixels (figure 4, equation (3.3)), we investigated the impact of drought and deforestation on fire patterns, not considering any political and economical variables that may influence fire dynamics in the region. We estimated that during the 2005 drought, the number of hot pixels (160 464 detections) were 43% higher than the expected value for a similar deforested area (approx. 19 000 km^2^). Using equation (3.3), we calculated the expected values under ‘normal’ and ‘dry’ conditions to estimate the impact of drought with increased deforestation on hot pixel counts. We found that the rate of hot pixel detection per kilometre square of deforested area annually would double under conditions similar to the 2005 drought. Moreover, the difference between the number of hot pixels in normal and dry conditions increases linearly with the increase of deforested area at a rate of 6.3 detections per kilometre square of deforested area annually (figure S2 in the electronic supplementary material). Based on these estimations, one can anticipate that the increased rate of hot pixel counts under drought conditions is likely to increase the area of forests affected by fires and consequently lead to the increase of CO_2_ emissions to the atmosphere due to biomass burning.

## 4. Conclusions

Our results stress a clear seasonality and synergic interaction between climate, deforestation and fires. We demonstrated here that anthropogenic forcing, such as land-use changes, is decisive in determining the seasonality and the annual patterns of fire occurrence. Moreover, drought events can increase significantly the number of fires in the region even with decreased deforestation rates. We may expect that the ongoing deforestation, currently based on slash and burn procedures, and the use of fires for land management in Amazonia will intensify the impact of droughts associated with natural climate variability or human-induced climate change, and therefore a large area of forest edges will be under increased risk of fire.

The impacts of fires on the Amazonian region could be reduced with the support of governments, since fires here are mainly induced by human activities and could be avoided and/or diminished by: the introduction of fire-free land management techniques; reinforcement of monitoring, controlling and application of the current Brazilian legislation to illegal fires; creation of protected areas (Nepstad *et al.* 2006); and environmental education programmes.

Some initiatives, such as the creation of the Extractive Reserves statutes, the Pilot Program to Conserve the Brazilian Rain Forest (PPG7), and Ecological–Economic Zoning (ZEE) project have been implemented in Brazil in attempts to approach the ideas of sustainable development and territorial planning in Amazonia (Alves 2008). On a smaller scale, the project ‘Roça sem Queimar’, meaning farming without the use of fires, led by a non-governmental organization in association with local farmers, is a pioneering experience that has been used as an alternative to the traditional slash and burn process in some Amazonian municipalities in the Xingu region (Silva *et al.* 2006).

Mechanized agriculture and crop plantations are not normally accompanied by subsequent fires (Eva & Lambin 2000), which can potentially reduce the risk of forest fires in Amazonia. Despite its negative ecological implications, this would be an important factor to be considered once the ZEE of the Amazonian region has been well stabilized. However, it is important to bear in mind that burning practices are common for some crops, such as sugar cane, which is largely used for biofuel production in Brazil.

Ultimately, this study showed that the time series used, including monthly deforestation data, provides a high temporal-resolution description of the interactions between land-use dynamics and climate. This information must be included in the current models for better understanding of the impact of climate change in the Amazonian ecosystems. However, intensive measurements of the carbon dynamics at fire-affected forests and the accurate mapping of the area and damage degree of burnt forests are still required for the total quantification of carbon emissions from fires in this region.

## Figures and Tables

**Figure 1:**
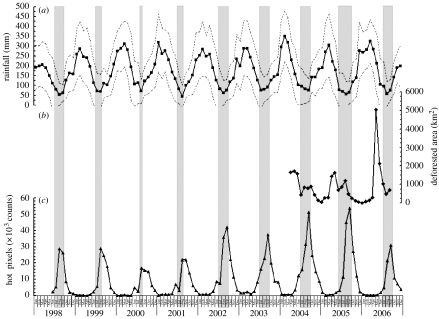
Monthly time series of (*a*) mean rainfall (mm) derived from the TRMM dataset (January 1998–December 2006), (*b*) cumulative deforested area (km^2^) from the INPE-DETER dataset (April 2004–September 2006) and (*c*) cumulative number of hot pixel detections from NOAA-12 dataset (May 1998–December 2006) within the limits of the Brazilian Legal Amazonia. Dashed lines in (*a*) correspond to the s.d. of the mean monthly rainfall (*n*=6705 pixels). Grey bars indicate the dry season length for each year (months with rainfall below 100 mm month^−1^).

**Figure 2:**
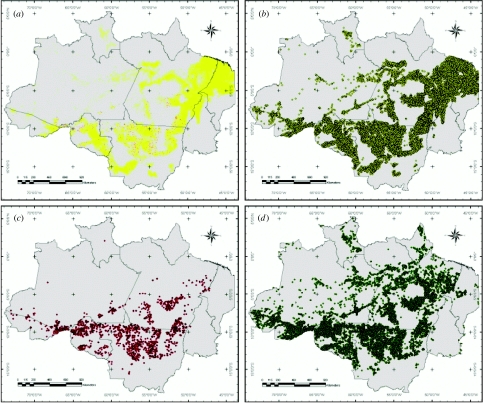
Maps of the Brazilian Amazonia showing (*a*) the total cumulative deforested area based on the INPE-DETER dataset until 2004 (yellow) and in 2005 (red), and the annual cumulative number of hot pixel detections in 2005 from NOAA-12 dataset over (*b*) areas deforested until 2004, (*c*) areas deforested in 2005 and (*d*) forested areas in 2005.

**Figure 3:**
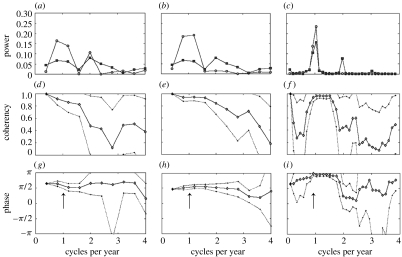
Spectral analysis on the monthly time series showing (*a*–*c*) the power spectra (Fourier periodograms) of (*a*) hot pixels and deforestation (*n*=30), (*b*) rainfall and deforestation (*n*=30) and (*c*) rainfall and hot pixels (*n*=103). The value at each temporal frequency gives the relative strength of the corresponding periodic component in each series. (*d*–*f*) The coherency spectra for the relationship between (*d*) hot pixels and deforestation, (*e*) rainfall and deforestation and (*f*) rainfall and hot pixels are shown. The value for each frequency gives the correlation (similar to the Pearson's correlation coefficient) between the corresponding periodic components in both signals. (*g*–*i*) The phase spectra between (*g*) hot pixels and deforestation, (*h*) rainfall and deforestation and (*i*) rainfall and hot pixels. Here, the values indicate the lag between the periodic components in both signals at each frequency (at one cycle yr^−1^, 2*π*, *π* and *π*/2 are equivalent to 12, 6 and 3 months time lag, respectively). The dashed lines in (*d*–*i*) indicate the 95% bilateral pointwise CI computed using a four-month smoothing window. The arrows indicate the phase shift for the coherent annual frequency. (*a*) Light grey circles, fires; dark grey squares, deforestation. (*b*) Light grey circles, rain; dark grey squares, deforestation. (*c*) Light grey circles, rain; dark grey squares, fires.

**Figure 4:**
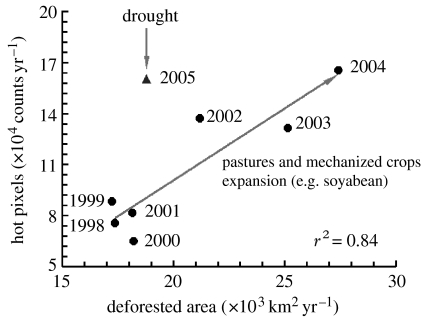
Linear regression between the annual cumulative number of hot pixels and the annual cumulative deforested area between 1998 and 2004 derived from the INPE-PRODES dataset (*n*=7, *p*=0.004). It shows the linear fit, indicated by the grey arrow and the coefficient of determination (*r*^2^). Note that 2005 is not included in the regression due to its anomalous characteristic as a function of the drought.
